# Maternal serum cadmium level during pregnancy and its association with small for gestational age infants: a population-based birth cohort study

**DOI:** 10.1038/srep22631

**Published:** 2016-03-03

**Authors:** Hua Wang, Lu Liu, Yong-Fang Hu, Jia-Hu Hao, Yuan-Hua Chen, Pu-Yu Su, Lin Fu, Zhen Yu, Gui-Bin Zhang, Lei Wang, Fang-Biao Tao, De-Xiang Xu

**Affiliations:** 1 School of Public Health, Anhui Medical University; Hefei, China; 2 Anhui Provincial Key Laboratory of Population Health & Aristogenics, Hefei, China

## Abstract

The association between maternal cadmium (Cd) exposure during pregnancy and the increased risk of fetal growth restriction (FGR) remains controversial. The present study evaluated the association between maternal serum Cd level and risk of small for gestational age (SGA) infants in a Chinese population. The present study analyzed a subsample of the C-ABCS cohort that recruited 3254 eligible mother-and-singleton-offspring pairs. Maternal serum Cd level during pregnancy was measured by graphite furnace atomic absorption spectrometry. The rate and odds ratio (*OR*) for SGA infant were calculated. The rate for SGA infant was 10.6% among subjects with H-Cd (≥1.06 μg/L), significantly higher than 7.5% among subjects with L-Cd (<1.06 μg/L). *OR* was 1.45 (95% *CI*: 1.11, 1.90; *P* = 0.007) among subjects with H-Cd. Adjusted *OR* for SGA infants was 1.43 (95% *CI*: 1.09, 1.88; *P* = 0.007) among subjects with H-Cd. Taken together, we observe the fact that maternal Cd exposure at middle gestational stage, elevates the risk of SGA in contrast to early gestational stage. The present results might be interesting and worth more discussing, and guarantee to further studies.

Cadmium (Cd) is an important occupational and environmental toxicant. Workers in electroplating, pigments, paints, welding, and Ni-Cd batteries are usually exposed to a high level of Cd^1^. As environmental Cd mainly distributed in soil, air and water, the general population is exposed to a low level of Cd via drinking water, food and cigarette smoking^2^. Cd is a male reproductive toxicant in humans. Numerous epidemiological investigations showed that environmental Cd exposure was associated with male infertility and poor semen quality in humans^3^,^4^,^5^,^6^,^7^. Cd is a testicular toxicant in rodent animals^8^. A lot of studies found that administration with a high dose of Cd induced germ cell apoptosis in mouse testes^9^,^10^,^11^,^12^,^13^,^14^. As an environmental endocrine disruptor, Cd could inhibit the synthesis of testosterone (T) through down-regulating key genes for T synthesis in testis^15^,^16^.

Increasing evidence demonstrates that Cd is an embryotoxic in rodent animals. Maternal Cd exposure during middle gestation induces fetal malformations in craniofacial region, limbs, trunk, viscera, and axial skeleton^17^,^18^,^19^,^20^,^21^,^22^, while mid- and late-gestation exposure induces fetal growth restriction (FGR) 16,23. Nevertheless, the association between maternal Cd exposure during pregnancy and adverse outcomes in women remains to be investigated.

FGR, including small for gestational age (SGA) infants, increases the risks of infant morbidity and metabolic diseases in adulthood^24^,^25^. The association between maternal Cd exposure during pregnancy and the increased risk of fetal growth restriction (FGR) remains controversial. An earlier report showed that fetal growth restriction is associated with higher placental Cd level^26^. According to recent mother-child cohort studies, maternal blood Cd level during pregnancy was associated with lower birth weight and the increased incidence of SGA^27^,^28^. Another prospective cohort study showed that maternal urinary Cd level during pregnancy was negatively associated with birth weight^29^. These results are in disagreement with those from other studies, in which there was no significant association between maternal Cd exposure during pregnancy and birth weight^30^,^31^. The present study mainly investigated the association between maternal serum Cd level during pregnancy and the risk of SGA infants in a large population-based birth cohort study. To explore the critical period for Cd-associated SGA, we further analyzed the association between maternal serum Cd level at different gestational stages and the risk of SGA infants in a Chinese population.

## Results

All alcohol drinkers and cigarette smokers during pregnancy were excluded from this study. Total 3254 mother-and-singleton-offspring pairs were eligible for this study. The demographic characteristics of pregnant women, including mother’s age, BMI before pregnancy, parity, and monthly income, were shown in Table 1. Total 1122 pregnant women (34.5%) were collected for serum in the first trimester and 2132 pregnant women (65.5%) were collected for serum in the second trimester. Median gestational age was 39 weeks (minimum: 31 weeks; maximum: 45 weeks). Among 3254 singleton infants, 82 were low birth weight infants and 336 were giant infants. In the present study, maternal serum Cd level was measured by graphite furnace atomic absorption spectrometry (GFAAS). The median serum Cd level was 0.79 μg/L (minimum: 0.04 μg/L; maximum: 8.08 μg/L). The 25^th^ and 75^th^ percentile was 0.57 and 1.06 μg/L. In addition, the distribution of maternal serum Cd levels during pregnancy and its association with birth weight were presented in Table 2. Total 601 (18.5%) pregnant women were for <0.50 μg/L, 2299 (70.7%) pregnant women for <1.00 μg/L, 2931 (90.1%) pregnant women for <1.50 μg/L. Interestingly, neonatal birth weight gradually decreased with the increase of maternal serum Cd level from 1.25 μg/L to more than 2.00 μg/L.

The association between maternal serum Cd level during pregnancy and fetal growth restriction was analyzed. As shown in Table 3, 7.5% newborns were with SGA infants among subjects with L-Cd. Interestingly, 10.6% newborns were with SGA infants among subjects with H-Cd (*OR*: 1.45; 95% *CI*: 1.11, 1.90; *P* = 0.007). After adjusting for pre-pregnancy BMI, maternal age, monthly income, gestational week for collecting serum, parity and gravidity, adjusted *OR* for SGA was 1.43 (95% *CI*: 1.09, 1.88; *P* = 0.009) among subjects with H-Cd using multiple logistic regression model (Table 3). According to a previous methods established by *Kippler et al.*, maternal serum Cd levels were classified as quartile 1 (Q1, <0.57 μg/L), quartile 2 (Q2, 0.57 to <0.79 μg/L), quartile 3 (Q3, 0.79 to <1.05 μg/L), and quartile 4 (Q4, ≥1.05 μg/L) base on quartiles 29. As shown in Table S1, 10.6% newborns were with SGA infants among subjects with Q4 (*OR*: 1.51; 95% *CI*: 1.07, 2.14; *P* = 0.020), significantly higher than 7.3% among subjects with Q1.

The association between maternal serum Cd level at early gestational stage and fetal growth restriction was analyzed. As shown in Table 4, 7.9% newborns were with SGA infant among subjects with L-Cd at early gestational stage. In addition, 9.4% newborns were with SGA infant among subjects with H-Cd at early gestational stage (*OR*: 1.20; 95% *CI*: 0.75, 1.93; *P* = 0.446). After adjusting for pre-pregnancy BMI, maternal age, monthly income, parity and gravidity, adjusted *OR* for SGA was 1.21 (95% *CI*: 0.75, 1.96; *P* = 0.432) among subjects with H-Cd at early gestational stage (Table 4).

The association between maternal serum Cd level at middle gestational stage and fetal growth restriction was analyzed. As shown in Table 5, 7.3% newborns were with SGA infants among subjects with L-Cd at middle gestational stage. Of interest, 11.2% newborns were with SGA infants among subjects with H-Cd at middle gestational stage (*OR*: 1.59; 95% *CI*: 1.15, 2.21; *P* = 0.006). After adjusting for pre-pregnancy BMI, maternal age, monthly income, parity and gravidity, adjusted *OR* for SGA was 1.57 (95% CI: 1.13, 2.19; *P* = 0.007) among subjects with H-Cd at middle gestational stage (Table 5).

## Discussion

Urinary Cd level is a good marker for Cd exposure^29^, but blood Cd level is proposed as a more accurate estimator of the accumulated body burden^32^,^33^. Although serum Cd level is only about 10% of blood Cd, it is well correlated with blood Cd level^34^. The present study used serum Cd level rather than blood Cd level as a marker for environmental Cd exposure during pregnancy allowing us much greater flexibility in collecting, storing and transporting samples. Several studies analyzed serum Cd level among non-occupational population. An earlier report showed that mean serum level was 0.46 μg/L among non-smoking women^35^. A report from Japan found that mean serum Cd level was 1.57 μg/L among women from a non-polluted area^36^. According to a small case-control study, maternal serum Cd level during pregnancy was 2.22 μg/L among subjects delivered with babies with normal birth weight and 3.33 μg/L among subjects delivered with babies with low birth weight^37^. A recent report from Korea showed that mean serum Cd level was 1.21 μg/L among non-occupational women^38^. The present study analyzed serum Cd level among 3254 pregnant women. We showed that median of maternal serum Cd level was 0.787 μg/L (range: 0.044 to 8.077 μg/L). At middle gestational stage, the median serum Cd level was 0.794 μg/L (range: 0.044 to 8.077 μg/L). As cigarette smoking is an important risk factor for FGR^39^,^40^ as well as associated with the increased serum Cd level^41^, cigarette smokers during pregnancy were excluded from the present study.

A recent study showed that maternal urinary Cd level was negatively associated with birth weight^29^. Another report from the EDEN mother-child cohort study found that maternal blood Cd level was negatively associated with birth weight in the offspring of women who smoked during pregnancy^28^. According to a recent report from a North Carolina cohort, high maternal blood Cd level during pregnancy was associated with the increased risk of SGA infants^27^. The present study analyzed the association between maternal serum Cd level during pregnancy and the risk of SGA infants among subjects in a Chinese population. We found that the incidence of SGA infants was 10.6% among subjects with H-Cd, significantly higher than 7.5% among subjects with L-Cd. The *OR* for SGA infants was 1.45 among subjects with H-Cd. After controlling for maternal age, pre-pregnancy BMI, monthly income, parity and gravidity, the adjusted *OR* for SGA infants was 1.43 among subjects with H-Cd. These results suggest that higher maternal serum Cd level during pregnancy was associated with the increased risk of SGA infants.

Several studies demonstrate that maternal Cd exposure at middle and late gestational stages induces FGR in mice^16^,^21^,^42^. However, a Canadian birth study found that no association was observed between Cd level in maternal blood from the first and third trimesters and risk for SGA infants^43^. Until now, no report analyzed the association between maternal Cd exposure at middle gestational stage and the risk of SGA infants. To investigate the critical period of Cd-associated SGA, the present study further compared the rate and *OR* of SGA infants among subjects with H-Cd at early and middle gestational stages. As expected, the rate of SGA infants was markedly increased among subjects with H-Cd at middle gestational stage. The *OR* was 1.59 among subjects with H-Cd at middle gestational stage. Unexpectedly, there was no significant difference on the rate of SGA infants between subjects with H-Cd at early gestational stage and subjects with L-Cd at early gestational stages. The *OR* was 1.20 (*P* > 0.05) among subjects with H-Cd at early gestational stage. These results suggest that maternal Cd exposure at middle gestational stage, but not early gestational stage, elevates the risk of SGA. The present results might be interesting and worth discussing, and guarantee to further studies.

The mechanism through which environmental Cd exposure during pregnancy results in FGR remains obscure. Two earlier studies observed the evidence of endoplasmic reticulum (ER) stress in human IUGR placentas 44,45. Data from animal experiments demonstrated that prolonged ER stress impaired not only placental development and morphogenesis but also placental transport and endocrine functions through down-regulating placental transporters and growth factors^44^,^46^. Indeed, a report from our laboratory found that maternal Cd exposure at middle gestational stage induced placental ER stress and impaired placental and fetal development in mice^21^. Therefore, we guess that placental ER stress, which impairs placental development and function, may be one of the main mechanisms for Cd-induced FGR. Additional study is necessary to explore the exact mechanism through which Cd-induced ER stress mediates placental dysfunction and FGR.

In the present study, we focused on the association of maternal Cd exposure during pregnancy and the risks of SGA infants. However, this study has some limitations. First, the present study did not investigate the effects of maternal exposure to other heavy metals, such as lead and mercury, on fetal development. Second, the present study did not analyze the association between maternal serum Cd level and risk for other adverse pregnancy outcomes. Third, the Cd exposure levels from two groups of pregnant women are not repeat measurements. Additional work is required to determine whether environmental lead and mercury exposure during pregnancy elevates the risks of SGA infants. In addition, whether maternal Cd exposure during pregnancy induces low birth weight (LBW) and preterm birth needs to be clarified.

In summary, we observe the fact that maternal Cd exposure at middle gestational stage, elevates the risk of SGA in contrast to early gestational stage. The present results might be interesting and worth more discussing, and guarantee to further studies.

## Methods

### Study population

The China-Anhui Birth Cohort Study (C-ABCS) is a prospective population-based cohort study that recruited 16 766 pregnant women from six major cities of Anhui province in China between November 2008 and October 2010. Exclusion criteria were as follows: inability to provide informed consent, alcohol drinking and cigarette smoking during pregnancy, mental disorders, pregnancy-induced hypertension and preeclampsia, gestational diabetes, heart disease, thyroid-related disease, a history of ≥3 previous miscarriages, or plans to leave local places before delivery^47^. The present study analyzed a subsample of the C-ABCS that recruited 4358 pregnant women from Hefei city of Anhui province from January 1 to December 31 in 2009. For this study, eligible participants were mother-and-singleton-offspring pairs in which serum samples from mothers were available for analysis of Cd level and offspring had a detailed birth records. Thirty-six pregnant women giving birth to twins, 15 fetal deaths, 2 stillbirths, 58 abortions and 589 withdrew were excluded from this study. In addition, 306 unavailable for maternal serum and 98 maternal serum collected at the third trimester were also excluded from this study. Total 3254 mother-and-singleton-offspring pairs were eligible for this study. All neonates were weighed at birth. The present study was approved by the ethics committee of Anhui Medical University. The methods were carried out in accordance with the approved guidelines. Oral and written consents were obtained from all pregnant women.

### Definition of SGA

According to the Global Reference^48^, we firstly calculated the average birth weight and standard deviation (SD) at 40 weeks of gestational age in the birth cohort. When the average birth weight and the coefficient of variation (12.31%, expressed as the percentage of SD to the mean birth weight) at 40 weeks of gestation for our population were entered into the Microsoft Office Excel Software in the webappendix 2, the programme generated the multiple reference percentiles of birth weight at 24 to 41 weeks of gestation (Table S2). A live-born infant with birth weight below 10^th^ percentile for the gestational age was defined as SGA infants in the present study.

### Measurement of serum Cd

Maternal fasting blood during pregnancy was collected in the morning. The blood samples were allowed to clot at room temperature for 30 min. Then maternal serum was obtained after centrifuging for 15 min at 3000 g. After discarding hemolytic specimens, available sera were stored at −80 °C until analysis. To avoid contamination of exogenous Cd, all centrifuge tubes, storage vials and transfer pipettes were soaked for at least 24 hr in 10% HNO_3_ at room temperature. Serum Cd concentration was determined by graphite furnace atomic absorption spectrometry (GFAAS; model: TAS-990; Purkinje General Instrument Co., Ltd, Beijing, China) coupled with a deuterium-lamp background correction system. All samples were prepared and analyzed according to a slightly modified method as previously described^16^. Serum samples were diluted with 1% HNO_3_ according to 1:4 (v/v). Matrix modifiers colloid palladium (Colpd^TM^, Xinda Measuring & Control Technology Co., Ltd, Chengdu, China) were added to each standard, blank and sample dilution. The above-mentioned mixture was then detected using GFAAS. Each sample was analyzed in triplicate. Precision of the method was measured by coefficients of variation. Mean CV for measurement of serum Cd was 5.16% for within-day determinations and 6.55% for day-to-day determinations. The limit of detection was 0.01 μg/L. In this study, all subjects were divided into two groups according to maternal serum Cd level during pregnancy as previously described^49^: low Cd group (L-Cd, <1.06 μg/L) and high Cd group (H-Cd, ≥1.06 μg/L) base on 75^th^ percentile.

### Confounding factors

According to a previous review^50^, potential confounding factors that might influence the association between maternal Cd exposure during pregnancy and SGA infants were chosen as follows: maternal age (≤24, 25–29 and ≥30 years), pre-pregnancy BMI (<18.5, 18.5 − 24.9 and ≥25 kg/m^2^), average monthly income (Low income for <2000 RMB or 312 US dollars per month; middle income for 2000–4000 RMB or 312–624 US dollars per month; high income for >4000 RMB or 624 US dollars per month), time for collecting serum (first trimester: median 11 weeks of gestation, range 4–12 weeks of gestation; second trimester: median 16 weeks of gestation, range 13–27 weeks of gestation), and parity (primiparae and multiparae).

### Statistical analysis

Proportions for maternal characters and neonatal characters in different serum Cd levels were provided. Differences between L-Cd group and H-Cd group were determined using chi-square tests. For adjustment of maternal age, pre-pregnancy BMI, monthly income, time for collecting serum and parity, Logistic regression model was used to estimate odds ratio (*OR*) and 95% confidence intervals (95% *CI*) for SGA infants. The forementioned results are referred to as relative risk (*RR*), because the *OR* is a good approximation of the risk ratio in the case of rare outcomes^51^. All statistical tests were two-sided using an alpha level of 0.05. We preformed all statistical analyses with statistical software *SPSS* (version 16.0).

## Additional Information

**How to cite this article**: Wang, H. *et al.* Maternal serum cadmium level during pregnancy and its association with small for gestational age infants: a population-based birth cohort study. *Sci. Rep.* **6**, 22631; doi: 10.1038/srep22631 (2016).

## Supplementary Material

Supplementary Information

## Figures and Tables

**Table 1:** Characteristics of 3254 mothers and their newborns.

Parameters	Maternal serum cadmium level^a^			*P-*value
	L-Cd (<*P*_75_, n = 2440)		H-Cd (≥*P*_75_, n = 814)	
*Maternal characteristics*				
Age [ *y*, n(*%*)]				
≤24		371 (15.2)	128 (15.7)	0.888
25–29		1540 (63.1)	515 (63.3)	
≥30		529 (21.7)	171 (21.0)	
Pre-pregnancy BMI [*kg/m*^*2*^, n(*%*)]				
<18.5		515 (21.1)	180 (22.1)	0.762
18.5–24.9		1848 (75.7)	611 (75.1)	
≥30		77 (3.2)	23 (2.8)	
Monthly income [n(*%*)]				
Low income^b^		1114 (45.7)	372 (45.7)	0.147
Middle income^b^		976 (40.0)	346 (42.5)	
High income^b^		350 (14.3)	96 (11.8)	
Parity [n(*%*)]				
Primiparae		2372 (97.2)	68 (2.8)	0.673
Multiparae		789 (96.9)	25 (3.1)	
Time for collecting serum [n(*%*)]				
First trimester		845 (34.6)	277 (34.0)	0.754
Second trimester		1595 (65.4)	537 (66.0)	
*Newborn characteristics*				
Sex [n(*%*)]				
Male		1310 (53.7)	427 (52.5)	0.542
Female		1130 (46.3)	387 (47.5)	
Gestational age [wk, n(*%*)]				
<37		87 (3.6)	94 (11.5)	<0.001
≥37		2353 (96.4)	720 (88.5)	
Birth weight (*g*, mean ± SD)		3413 ± 443.8	3363 ± 510.6	0.013

^a^L-Cd for serum cadmium <1.06 μg/L, and H-Cd for serum cadmium ≥1.06 μg/L.

^b^Low income for <2000 RMB (312 US dollars) per month; middle income for 2000–4000 RMB (312–624 US dollars) per month; high income for >4000 RMB (624 US dollars) per month.

**Table 2:** The distribution of maternal serum Cd levels during pregnancy and its association with birth weight.

Serum Cd (μg/L)	n	Cumulative Percentage (%)	Birth Weight (g, 95%*CI*)
<0.25	140	4.3	3415.6 (3343.8, 3487.3)
0.25 – 0.49	461	18.5	3441.2 (3395.6, 3486.8)
0.50 – 0.74	872	45.3	3399.9 (3371.4, 3428.4)
0.75 – 0.99	826	70.7	3405.3 (3376.3, 3434.2)
1.00 – 1.24	418	83.5	3408.0 (3360.2, 3455.8)
1.25 – 1.49	214	90.1	3411.3 (3348.4, 3474.2)
1.50 – 1.74	139	94.3	3317.0 (3220.5, 3413.5)
1.75 – 1.99	145	98.8	3309.5 (3225.8, 3393.2)
≥2.00	39	100.0	3292.0 (3129.5, 3454.5)

**Table 3:** The incidence and odds ratio (*OR*) for SGA infants based on maternal serum Cd level during pregnancy.

	Maternal serum Cd level^a^		*P-*value
	Low (<*P*_75_)	High (≥*P*_75_)	
Number of live infants	2440	814	–
Number of SGA	184	86	–
Incidence (*%*)	7.5	10.6	0.007
Univariate *OR* (95%*CI*)	1.00	1.45 (1.11, 1.90)	0.007
Adjusted *OR* (95%*CI*)^b^	1.00	1.43 (1.09, 1.88)	0.009

^a^Low for serum cadmium <1.06 μg/L, and High for serum cadmium ≥1.06 μg/L.

^b^Adjusted for pre-pregnancy BMI, maternal age, time for collecting serum, monthly income, parity and gravidity.

**Table 4:** The incidence and odds ratio (*OR*) for SGA infants based on maternal serum Cd level in the first trimester.

	Maternal serum Cd level^a^		*P-*value
	Low (<*P*_75_)	High (≥*P*_75_)	
Number of live infants	845	277	–
Number of SGA	67	26	–
Incidence (*%*)	7.9	9.4	0.445
Univariate *OR* (95%*CI*)	1.0	1.20 (0.75, 1.93)	0.446
Adjusted *OR* (95%*CI*)^b^	1.0	1.21 (0.75, 1.96)	0.432

^a^Low for serum cadmium <1.06 μg/L, and High for serum cadmium ≥1.06 μg/L.

^b^Adjusted for pre-pregnancy BMI, maternal age, and monthly income, parity and gravidity.

**Table 5:** The incidence and odds ratio (*OR*) for SGA infants based on maternal serum Cd level in the second trimester.

	Maternal serum Cd level^a^		*P-*value
	Low (<*P*_75_)	High (≥*P*_75_)	
Number of live infants	1595	537	–
Number of SGA	117	60	–
Incidence (*%*)	7.3	11.2	0.005
Univariate *OR* (95%*CI*)	1.00	1.59 (1.15, 2.21)	0.006
Adjusted *OR* (95%*CI*)^b^	1.00	1.57 (1.13, 2.19)	0.007

^a^Low for serum cadmium <1.06 μg/L, and High for serum cadmium ≥1.06 μg/L.

^b^Adjusted for pre-pregnancy BMI, maternal age, and monthly income, parity and gravidity.
