# Supplementary material for: Maternal serum cadmium level during pregnancy and its association with small for gestational age infants: a population-based birth cohort study
Source: Sci Rep. 2016 Mar 3;6:22631. doi: 10.1038/srep22631 (PMC4776171; doi:10.1038/srep22631)
Supplement: Supplementary Information [file srep22631-s1.doc]

Maternal serum cadmium level during pregnancy and its association with small for gestational age infants: a population-based birth cohort study

Hua Wang^1,2*^, Lu Liu^1*^, Yong-Fang Hu^1^, Jia-Hu Hao^1, 2^, Yuan-Hua Chen ^1, 2^, Pu-Yu Su^1, 2^, Lin Fu^1^, Zhen Yu^1^, Gui-Bin Zhang^1^, Lei Wang^1^, Fang-Biao Tao^1,2^, De-Xiang Xu^1,2^

^1^School of Public Health, Anhui Medical University; ^2^Anhui Provincial Key Laboratory of Population Health & Aristogenics, Hefei, China

*These authors contributed equally to this work.

Address correspondence to: Prof. De-Xiang Xu, School of Public Health, Anhui Medical University, Hefei 230032, China; Tel: +86 551 65167923; Email: xudex@126.com; Prof. Fang-Biao Tao, School of Public Health, Anhui Medical University, Hefei 230032, China; Tel: +86 551 65161168; Email: fbtao@126.com.

**Table S1 The incidence and odds ratio (*OR***) for SGA infants based on maternal serum Cd level during pregnancy

|  | Maternal serum Cd level ^a^ | | | | *P* |
| --- | --- | --- | --- | --- | --- |
|  | Q1 | Q2 | Q3 | Q4 |  |
| Number of live infants | 813 | 814 | 813 | 814 | － |
| Number of SGA | 59 | 73 | 52 | 86 | － |
| Incidence (*%*) | 7.3 | 9.0 | 6.4 | 10.6 | 0.012 |
| Univariate *OR* (95%*CI*) | 1.00 | 1.26 (0.88, 1.80) | 0.87 (0.59, 1.29) | 1.51 (1.07, 2.14) ^c^ | 0.012 |
| Adjusted *OR* (95%*CI*) ^b^ | 1.00 | 1.22 (0.85, 1.75) | 0.85 (0.58, 1.25) | 1.46 (1.03, 2.07) ^c^ | 0.016 |

^a^ According to a previous methods established by *Kippler et al.,* maternal serum Cd levels were classified as quartile 1 (Q1, <0.57 μg/L), quartile 2 (Q2, 0.57 to <0.79 μg/L), quartile 3 (Q3, 0.79 to <1.05 μg/L), and quartile 4 (Q4, ≥ 1.05 μg/L) base on quartiles.

^b^ Adjusted for pre-pregnancy BMI, maternal age, gestational week for collecting serum, monthly income, parity and gravidity.

^c^ *P*＜0.05, as compared with Q1.

| **Table S2 Percentiles of birth weight for a population with the mean birthweight**  **at 40 weeks of gestation of 3511.6 g in China ^a^** | | | | | | | | | | | |
| --- | --- | --- | --- | --- | --- | --- | --- | --- | --- | --- | --- |
| **Gestational age (wk)** | **Birth weight percentiles** | | | | | | | | | | |
|  | **99th** | **97th** | **95th** | **90th** | **75th** | **mean** | **25th** | **10th** | **5th** | **3rd** | **1st** |
| **24** | 874 | 839 | 820 | 791 | 742 | 688 | 634 | 585 | 556 | 537 | 501 |
| **25** | 1021 | 979 | 957 | 923 | 866 | 803 | 740 | 683 | 649 | 627 | 585 |
| **26** | 1184 | 1135 | 1110 | 1070 | 1004 | 931 | 858 | 792 | 752 | 727 | 678 |
| **27** | 1363 | 1307 | 1278 | 1232 | 1156 | 1072 | 988 | 912 | 866 | 837 | 781 |
| **28** | 1558 | 1494 | 1461 | 1409 | 1322 | 1225 | 1129 | 1042 | 990 | 957 | 893 |
| **29** | 1768 | 1696 | 1658 | 1599 | 1500 | 1391 | 1281 | 1183 | 1124 | 1086 | 1013 |
| **30** | 1993 | 1912 | 1868 | 1802 | 1691 | 1568 | 1444 | 1333 | 1267 | 1224 | 1142 |
| **31** | 2230 | 2139 | 2091 | 2017 | 1892 | 1754 | 1616 | 1492 | 1418 | 1369 | 1278 |
| **32** | 2478 | 2377 | 2323 | 2241 | 2103 | 1949 | 1796 | 1658 | 1575 | 1522 | 1420 |
| **33** | 2735 | 2623 | 2564 | 2472 | 2320 | 2151 | 1982 | 1829 | 1738 | 1679 | 1567 |
| **34** | 2996 | 2873 | 2809 | 2709 | 2542 | 2356 | 2171 | 2004 | 1904 | 1839 | 1717 |
| **35** | 3259 | 3126 | 3055 | 2947 | 2765 | 2563 | 2362 | 2180 | 2072 | 2001 | 1868 |
| **36** | 3520 | 3377 | 3300 | 3183 | 2987 | 2769 | 2551 | 2355 | 2238 | 2161 | 2017 |
| **37** | 3776 | 3621 | 3540 | 3414 | 3204 | 2970 | 2736 | 2526 | 2400 | 2318 | 2164 |
| **38** | 4021 | 3857 | 3770 | 3636 | 3412 | 3163 | 2914 | 2690 | 2556 | 2469 | 2304 |
| **39** | 4252 | 4078 | 3986 | 3845 | 3608 | 3345 | 3081 | 2845 | 2703 | 2611 | 2437 |
| **40** | 4465 | 4282 | 4186 | 4037 | 3788 | 3512 | 3236 | 2987 | 2838 | 2741 | 2559 |
| **41** | 4655 | 4465 | 4364 | 4209 | 3950 | 3661 | 3373 | 3114 | 2959 | 2858 | 2668 |

^a^ Data obtained from the calculation sheet for Microsoft Office Excel software (webappendix B) according to the previous literature (*Mikolajczyk*. et al, 2011). Standard deviation of 12.31% of the corresponding mean birthweight was used for calculation of percentiles.
